# Comparison of Cost-Utility, Visual Acuity, and Humanistic Outcomes of Cataract Surgery Performed in the United States Versus International Outreach Campaigns

**DOI:** 10.3390/jcm14093037

**Published:** 2025-04-28

**Authors:** Lauren E. Chen, Ryka Vahidi, Anna Kesler-Diaz, Sameh Mosaed

**Affiliations:** 1 Department of Ophthalmology, Gavin Herbert Eye Institute, University of California Irvine, Irvine, CA 92617, USA; smosaed@hs.uci.edu; 2 University of California Los Angeles, Emergency Medical Services, Los Angeles, CA 90095, USA; ryka04@ucla.edu; 3 Department of Ophthalmology, Palo Alto Medical Foundation, Santa Cruz, CA 95060, USA; akeslerdiaz@gmail.com

**Keywords:** phacoemulsification, international outreach, quality of life

## Abstract

**Background:** This study compares the cost-utility, visual acuity, and humanistic outcomes of phacoemulsification surgery performed during international outreach campaigns to outcomes in the U.S. **Objective**: Humanitarian organizations frequently perform cataract surgeries in underserved regions, yet the outcomes of phacoemulsification in these settings remain unexplored. This study aims to compare the visual acuity outcomes, quality-of-life outcomes, and cost of phacoemulsification surgery during international outreach campaigns with those in the U.S. **Methods**: This retrospective review analyzed 344 cases from Women for World Health (W4WH) missions and 305 consecutive cases from the academic practice of the lead surgeon of W4WH in the US. The pre-operative (pre-op) visual acuity, post-operative (post-op) visual acuity at day 1, and best-corrected visual acuity (BCVA) at post-op month 1 were compared to baseline. The change in visual acuity was compared between the two groups. Complication rates, cost per case, and quality-of-life measures were evaluated for the mission group compared to cataract surgery outcomes in the developed world. **Results**: The mission cohort had worse baseline visual acuity than the U.S. cohort (1.36 logMAR vs. 0.50 logMAR, *p* < 0.0001) and showed greater mean improvement (1.24 logMAR vs. 0.35 logMAR, *p* < 0.0001). Both cohorts achieved similar BCVA at post-op month 1 (0.13 logMAR). The cost for the mission group was USD 276 per surgery. A total of 93.94% of patients in the mission group achieved a BCVA of 20/40 or better, which is comparable to the U.S. outcomes. **Conclusions**: The dramatic impact, immediacy, and enduring effect of cataract surgery using phacoemusification on quality of life, mental health, and economic return on investment make it the ideal procedure for humanitarian settings. Considering all of the available metrics, our data support the continued expansion of humanitarian efforts by cataract surgery organizations worldwide.

## 1. Introduction

The number of people with severe vision loss and blindness secondary to cataracts has increased over the past 30 years, making cataracts the number one cause of reversible blindness in the world [1]. In 2020, cataracts accounted for over 15 million cases of avoidable blindness among adults aged 50 and older, representing approximately 45% of global blindness in this age group [1]. Cataract surgical rate (CSR) is defined as the number of cataract operations performed per million individuals per year and is considered an indicator of access to eye care [1]. Globally, CSR is highly correlated to economic indicators, such as gross domestic product per capita and gross national income per capita, highlighting disparities in surgical access [2,3,4,5]. The primary factor found to be influencing CSR is the economic status of the countries [4]. Population growth and aging have led to cataract rates outpacing the treatment programs aimed at managing them, making global surgical outreach programs vital in underserved areas [6]. Many different surgical mission organizations conduct missions around the globe to these underprivileged communities. These organizations rely on intensive time commitments from highly trained volunteer surgeons, nurses, coordinators, and ancillary personnel. These missions require heavy front-loading of efforts for gathering of equipment, supplies, site evaluations, coordinating with local host organizations, patient pre-op evaluation, and plans for ongoing post-op care, if any. The input of significant time, personal cost, and effort from involved individuals during cataract surgery missions has yet to be evaluated from visual outcomes, humanistic (“quality of life”—QOL), and cost-analysis perspectives. This study aims to analyze the visual acuity and humanistic outcomes, as well as cost comparisons, of cataract surgeries performed during international surgical missions compared with outcomes of those performed in the U.S.

## 2. Methods

We retrospectively reviewed the pre-op and post-op records from surgeries performed on humanitarian mission trips from May 2018 to February 2025 from a single non-profit charity surgical organization, Women for World Health. These surgeries were performed in various cities in Peru, Ecuador, and Bolivia. The cataract surgeries for the mission cohort were performed on the Oertli Catarhex 3 portable phacoemulsification unit (Berneck, Switzerland), obtained on loan from SEE International (Santa Barbara, CA, USA). A retrospective chart review was conducted of the past 305 consecutive cataract cases, performed by the lead surgeon of Women for World Health in the U.S. at a single academic medical center between 15 March 2021 and 10 December 2024, and not performed in combination with any other surgery (Gavin Herbert Eye Institute, University of California, Irvine, CA, USA). These procedures were performed using the Stellaris phacoemulsification system (Bausch and Lomb, St. Louis, MO, USA). Snellen visual acuity was converted to the logMAR scale. The BCVA pre-op was compared to uncorrected visual acuity (UCVA) post-op day 1, as well as BCVA post-op month 1 for both groups using an unpaired, two-tailed student’s *t*-test. The mean logMAR visual acuity improvement was compared between the two groups using an unpaired, two-tailed student’s *t*-test. The percentage of patients regaining better than 20/200 from a baseline worse than 20/200 was calculated and compared for the two groups. The percentage of patients regaining a visual acuity of better than 20/40 from a baseline of worse than 20/40 was also calculated and compared for the two groups. The BCVA at post-op month one for patients in the mission group for whom such data were available was also collected, and the percentage of patients achieving a BCVA of better than 20/40 was calculated for this group. The BCVA for the U.S. group at post-op month one for the same corresponding timeframe was collected for analysis. Demographic data, such as age, sex, and laterality, were tabulated. Cost per surgery was also calculated for the mission group. Complication rates (defined as posterior capsular rupture, anterior capsular tear, vitreous loss, and any lens implantation outside the capsular bag) were calculated for the two groups. The study was conducted in accordance with the Declaration of Helsinki and approved by the Institutional Review Board of the University of California, Irvine (protocol #20195254, approval date 4 March 2025).

## 3. Results

A total of 376 consecutive cases from the Women for World Health were reviewed. Thirty two cases were excluded, as these patients underwent combined glaucoma surgery or failed to present for the follow-up on post-op day 1, resulting in 344 cases. A total of 305 consecutive cases of the lead surgeon from W4WH from a U.S.-based tertiary-care academic center were reviewed. There were 198 patients in the mission group for which post-op month 1 data were available, including BCVA. Baseline demographics for both groups are summarized in Table 1. The patients in the mission group were statistically significantly younger (68.16 years vs. 75.5 years, *p* < 0.0001). The mean pre-op visual acuity was significantly worse in the mission group (1.36 logMAR vs. 0.5 logMAR, *p* < 0.0001).

**Figure 1:**
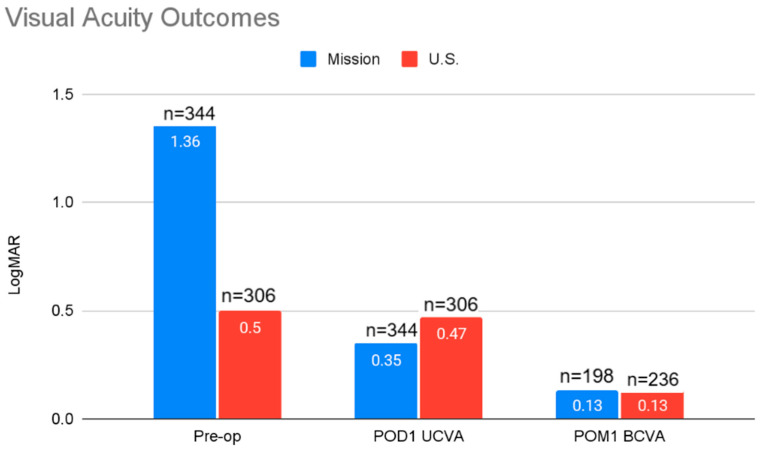
Visual-acuity outcomes for the mission and U.S. groups.

Visual acuity outcomes and comparisons are summarized in Figure 1. The mean logMAR visual acuity in the mission group post-op day 1 was 0.4 (*n* = 344) and BCVA at post-op month one was 0.13 logMAR (*n* = 198). The mean logMAR visual acuity in the U.S. group post-op day 1 was 0.35 (*n* = 305) and BCVA at post-op month one was 0.13 logMAR (*n* = 235).

**Figure 2:**
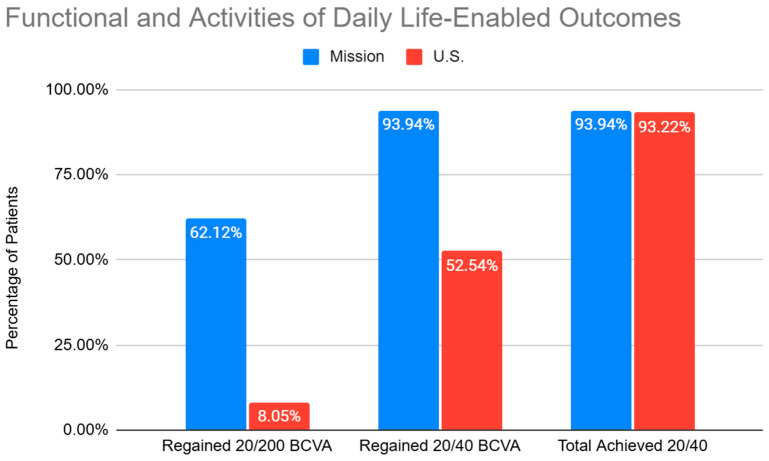
Quality-of-life outcomes for the mission and U.S. groups.

The functional visual acuity outcomes are summarized in Figure 2. A total of 62.12% of the patients in the mission group regained functional vision of 20/200 or better from a baseline of worse than 20/200, and 93.94% regained an UCVA of 20/40 or better from a baseline of worse than 20/40. At post-op month one, 93.94% of patients in the mission group achieved a BCVA of 20/40 or better, which was statistically similar to the U.S. cohort, achieving a BCVA of 20/40 or better at 93.22%. In the U.S. cohort, 8.05% regained functional vision of 20/200 or better from a baseline of worse than 20/200, and 52.54% regained vision of 20/40 or better from a baseline of worse than 20/40. The mission group had a mean improvement in visual acuity of 0.88 logMAR vs. 0.15 logMAR in the U.S. group. The improvement in visual acuity between the mission group and the U.S. group was statistically significant (*p* < 0.0001). The mission group had a complication rate of 0.58%, compared to 0% in the U.S. group. The cost for the mission group was USD 276 per surgery. The raw data supporting the conclusions of this article will be made available by the authors upon request.

## 4. Discussion

While there are many active cataract surgical mission organizations performing surgery in underserved countries worldwide, there are limited data in the literature supporting this activity from outcomes and cost perspectives. This study aimed to quantify and compare the costs, visual acuity improvement, and humanistic outcomes from a humanitarian cataract surgical organization to outcomes typically encountered in the U.S. by similar surgeons with advanced surgical experience. 

Our data show that greater vision recovery was seen among the mission cohort than among the U.S. cohort. This significant difference is largely attributed to worse mean baseline pre-op visual acuity in the mission group compared to the U.S. group. The threshold crossed for the re-acquisition of functional vision was far greater in the mission patient population. We defined the threshold for functional vision as 20/200 or better, as the definition of legal blindness in the U.S. is vision worse than 20/200 in the better-seeing eye. Several studies have found that for mobility tasks, most persons were not disabled until they had significant acuity loss (logMAR visual acuity > 1.0 or <20/200) [7]. From a QOL perspective, 53% of patients regained functional vision from a baseline worse than 20/200 in a humanitarian setting compared to 7.79% in the U.S. group. This is the result of more patients experiencing declining vision beyond the definition of legal blindness prior to cataract surgery in the mission group, and it underscores the need for ongoing and expanded humanitarian efforts in underserved areas. We also evaluated the percentage of patients regaining adequate visual acuity to carry out the instrumental activities of daily life, such as driving (20/40 or better), from a baseline of worse than 20/40. A total of 41.57% of the patients in the mission group regained this acuity without spectacle correction, while for those with a refraction at post-op month one, this number increased to 93.94%. This threshold of vision has been found to be the best discriminator between subjects with or without limitations in instrumental activities of daily life (global, physical, and cognitive) [8]. This underscores the crucial activities of daily life that can be resumed through interventions provided during humanitarian missions, often allowing patients to re-enter the workforce and liberating caregivers to also re-engage with employment and other vital life activities. This study does not suggest superior outcomes of mission campaigns but rather highlights that similar post-operative visual acuities can be achieved regardless of the baseline visual acuity if a cataract is the main cause of vision loss. 

From a cost-analysis perspective, the cost utility of expanded humanitarian surgical missions is supported by our data. Supplies, such as viscoelastic material, blades, sutures, sterile supplies, and intraocular lenses, have widely varying costs depending on where the supplies are sourced, and, hence, the cost of each surgery can vary widely. However, for our organization, the cost for each surgery—including transportation and accommodation costs for the team members, reusable equipment, and disposable supplies—was calculated to be USD 276. It should be noted that some of the supplies were donated to our organization through other non-profit groups that provide support to cataract mission organizations in the U.S. Additionally, we used the portable Catarhex3 phacoemulsification unit that is available on loan from many mission organizations and, therefore, did not incur costs of purchasing the unit directly. This cost would not be relevant during missions where manual small incision cataract surgery (MSICS) is employed. Indeed, MSICS has been shown to be a time- and cost-efficient surgical technique, especially for hyper mature cataracts, which are more prevalent in a mission setting [9]. If the phacoemulsification unit were to have been purchased, it would have added USD 87 to each case, but this cost per case would decrease as the unit would be used for more cases going forward. Only topical anesthesia was used during the missions, obviating the need for the anesthesia personnel and related costs that are standard in the U.S. Intravenous lines are typically not placed for cataract surgery in mission settings, decreasing the cost of supplies and personnel. Additionally, most mission organizations waste fewer unused medications and sterile supplies than are routinely discarded per case in the U.S. For example, a single bottle of topical anesthetic (e.g., tetracaine) can be used to provide robust topical anesthesia for at least 20 patients in a mission setting. In resource-rich environments, one bottle per patient is the standard utilization. According to the Center for Medicare Services claims data from 2019, the average cost of cataract surgery performed in a hospital outpatient surgery center in the U.S. for Medicare beneficiaries was USD 2627. This included the surgeon’s fee of USD 548 and the facility’s fee of USD 2079 [9]. A recent analysis by Brown et al. (2018) found that, even at this cost, a first-eye cataract surgery resulted in a 2.523 quality-adjusted life-year (QALY) gain, a 33.3% patient value gain, and a 25.5% quality-of-life gain [10]. The authors concluded that cataract surgery performed in the U.S., when analyzed by standard health economic methodologies, is highly cost-effective. In comparison, our data show a roughly 10-fold decrease in the cost of performing the same surgery with similar outcomes in a younger patient population in a humanitarian mission setting. It follows that the return on investment (ROI) in terms of quality of life and QALY is magnitudes higher in underserved areas. The reverberative effects of this procedure on national wealth and GDP cannot be underestimated, as it allows patients and caregivers to reintegrate into gainful employment and societal productivity.

The complication rates experienced in the U.S. cohort was 0%, compared to 0.58% for the mission cohort. Two cases of posterior capsular rupture were encountered, with one case involving retained nuclear fragments and placement of an anterior chamber intraocular lens, requiring referral to the nearest ophthalmic surgical facility for vitrectomy. The other case was managed with manual dry vitrectomy and placement of a sulcus intraocular lens with a good post-operative outcome. This complication rate is similar to complication rates for cataract surgeries performed by high-volume cataract surgeons in developed countries [11,12]. Low complication rates were experienced despite the mission cohort consisting mostly of higher density, traumatic, and white cataracts. These types of cataracts are known to increase complication rates during phacoemulsification [13,14]. While complications can increase the cost of surgery by necessitating further interventions, the incidence is very low and does not appreciably impact the overall budgets of such campaigns. 

Our data show that the average age of patients in the mission cohort was 68.16 years compared with the average age of cataract extraction in the U.S. cohort at 75.50 years. The development of severe cataracts at a younger age may have many etiologies: untreated medical conditions that exacerbate cataract development, such as diabetes, poor nutrition, ultraviolet light exposure, smoking, ocular trauma associated with occupational trauma, and lack of access to ophthalmic care for ocular comorbidities that promote cataract development such as uveitis [15,16,17,18].

In addition to improving the socioeconomic status of individuals and their communities, cataract surgery has been shown to decrease anxiety and depression, as well as improve overall mental health [19]. Another study by Hodge et al. demonstrated that a delay in timely access to cataract surgery increases the risk of falls, dependance on caregivers, and decreases overall quality of life [20]. The benefits of cataract surgery on the mental health, secondary health outcomes, and overall quality of life in younger populations endure over a patient’s lifetime, as cataracts do not recur or require maintenance treatment. The impact of a “one-and-done” procedure like cataract surgery makes it an optimal procedure for humanitarian organizations to prioritize. 

There are several limitations to our findings. Firstly, these data do not adequately reveal the full extent of vision recovery in either group, as typical cataract surgery patients require at least one month for full stabilization of vision following the resolution of corneal edema and anterior segment inflammation. As typical follow-up for patients during a humanitarian mission campaign does not extend beyond post-op day 1, this is often the extent of the follow-up available for such an analysis. The patients in the mission cohort are expected to have much worse corneal edema on post-op day 1, as the density of the cataracts are directly proportional to the ultrasound energy required to emulsify the cataract and remove it through the method of phacoemulsification [21]. We fully expect that this analysis greatly underestimates the visual recovery of the patients in the mission cohort for which only post-op day 1 data were available. A total of 198 patients in our mission cohort underwent surgery then followed up in a clinic where a local ophthalmologist was available to provide ongoing care. The data from this group of nearly 200 patients reveal that 94% of patients achieved a final Snellen visual acuity of better than 20/40 at post-op month one. This represents a greater than eight-line improvement in visual acuity and highlights the gains in vision that can be expected after a brief recovery period and spectacle correction. 

Phacoemulsification is the standard method of cataract extraction in the U.S., and it requires the use of highly specialized equipment that is both costly and difficult to readily transport. For these reasons, many mission organizations utilize the manual method of cataract extraction, known as manual small-incision cataract surgery (MSICS). This method does not require expensive technology and does not increase post-operative corneal edema but does involve making a larger incision to manually express the cataract in toto. This method of cataract extraction is known to be more cost-effective than phacoemulsification [22,23]. Our aim was to directly compare outcomes of surgery performed during missions to the standard-of-care in developed countries using the same technology. Hence, our vision outcomes and cost analysis cannot be applied to other organizations that may acquire their supplies from different sources or employ different methods of cataract extraction.

Another limitation of our study is that many patients in our humanitarian mission cohort did not have pre-op keratometry and biometry calculations to optimize visual outcomes by reducing refractive errors. In these cases, average IOL powers were implanted with the hope that spectacle correction could be employed for visual optimization. The post-op day 1 acuities do not reflect BCVA results that would be obtained with appropriate IOL power and spectacle correction. All patients in the U.S. cohort did have reliable IOL calculations based on biometry and keratometry performed by trained personnel, and, thus, post-op day 1 UCVA in this cohort will be as optimal as possible. The importance of accurate biometry and keratometry during mission surgeries was highlighted in another study by Urbinati et al. [24]. Lastly, many patients in the humanitarian mission cohort may have had undiagnosed retinal or optic nerve pathologies that could not be properly assessed preoperatively because of the density of the cataracts. This contrasts with the patients operated on at the U.S. academic medical center, where a full ophthalmic evaluation is conducted preoperatively with referral to subspecialists, if warranted, to accurately predict visual potential and optimize all other ocular comorbidities prior to cataract surgery. Patients with ocular comorbidities such as glaucoma, diabetic retinopathy, or macular degeneration were not excluded from the analysis in an effort to keep both cohorts as similar as possible such that the mission cohort did not have detailed pre-op retinal examinations. As the patients in the U.S. cohort were derived from the practice of a subspecialist in an academic tertiary referral center, we expect considerable ocular comorbidities, and, thus, the results may not be comparable to the isolated cataract surgery data in the literature. Indeed, our results reflect real-world experience with diverse patient populations with a myriad of comorbid ocular pathologies. 

While the inputs of expertise, time, manpower, planning, and personal expense by the volunteer team members must be considered as costs during a humanitarian mission, these are intangible and impossible to quantify. Conducting cataract surgical missions using phacoemulsification techniques yield superb visual acuity results, similar complication rates as those encountered in developed countries, lower costs per case than in resource-rich countries, and considerable QOL improvements for patients in these underserved areas. The dramatic impact on visual acuity, the immediacy in which this impact is attained, and the enduring effect of this intervention make cataract surgery the ideal procedure for humanitarian settings. Considering all the available metrics, our data support the continued expansion of humanitarian efforts by cataract surgery organizations worldwide.

## Figures and Tables

**Table 1:** Baseline demographic data for the mission and U.S. groups.

	Mission	U.S.	*p*-Value
Number of cases (*n*)	344	305	
Timeframe	May 2018–February 2025	15 March 2021–10 December 2024	
Phacoemulsification unit	Portable Oertli Catarhex 3 (Berneck, Switzerland)	Stellaris (Bausch and Lomb, St. Louis, MO, USA)	
Male	148	131	(*p* = 1.0)
Female	196	174	(*p* = 1.0)
Age	68.16	75.5	**(*p* < 0.0001)**
OD	173	157	(*p* = 0.857)
OS	171	149	(*p* = 0.857)
Mean pre-op BCVA (logMAR)	1.36	**0.5**	**(*p* < 0.0001)**

(Boldface values are any significant *p* values < 0.05).

## Data Availability

The raw data supporting the conclusions of this article will be made available by the authors on request.
